# Involvement of P2X7 receptor in neuronal degeneration triggered by traumatic injury

**DOI:** 10.1038/srep38499

**Published:** 2016-12-08

**Authors:** Francisco M. Nadal-Nicolás, Caridad Galindo-Romero, Francisco J. Valiente-Soriano, María Barberà-Cremades, Carlos deTorre-Minguela, Manuel Salinas-Navarro, Pablo Pelegrín, Marta Agudo-Barriuso

**Affiliations:** 1 Instituto Murciano de Investigación Biosanitaria-Virgen de la Arrixaca (IMIB-Arrixaca), Murcia, Spain; 2 Departamento de Oftalmología, Facultad de Medicina. Universidad de Murcia, Murcia, Spain; 3 Unidad de Inflamación Molecular y Cirugía Experimental, Hospital Clínico Universitario Virgen de la Arrixaca, Murcia, Spain; 4 Centro de Investigación Biomédica en Red en el Área temática de Enfermedades Hepáticas y Digestivas (CIBERehd), Spain

## Abstract

Axonal injury is a common feature of central nervous system insults that culminates with the death of the affected neurons, and an irreversible loss of function. Inflammation is an important component of the neurodegenerative process, where the microglia plays an important role by releasing proinflammatory factors as well as clearing the death neurons by phagocytosis. Here we have identified the purinergic signaling through the P2X7 receptor as an important component for the neuronal death in a model of optic nerve axotomy. We have found that in P2X7 receptor deficient mice there is a delayed loss of retinal ganglion cells and a decrease of phagocytic microglia at early times points after axotomy. In contralateral to the axotomy retinas, P2X7 receptor controlled the numbers of phagocytic microglia, suggesting that extracellular ATP could act as a danger signal activating the P2X7 receptor in mediating the loss of neurons in contralateral retinas. Finally, we show that intravitreal administration of the selective P2X7 receptor antagonist A438079 also delays axotomy-induced retinal ganglion cell death in retinas from wild type mice. Thus, our work demonstrates that P2X7 receptor signaling is involved in neuronal cell death after axonal injury, being P2X7 receptor antagonism a potential therapeutic strategy.

Damage to the optic nerve (ON), formed by the axons of retinal ganglion cells (RGCs) neurons, causes their retrograde death and the consequent loss of vision. In humans, optic nerve trauma can be caused by head injury or growing tumors. RGC loss also occurs in retinal pathologies that have a component of the ON such as glaucoma^1^, or optic nerve diseases, such as Leber’s hereditary optic neuropathy. Axonal injury also occurs in many other forms of central nervous system insults, such as stroke or traumatic brain injury^2^.

Optic nerve axotomy is a very well characterized and reproducible model of CNS axonal damage. In mice, RGC death after ON axotomy is already significant at day 3, and stabilizes at day 9, when around 20% of the original RGC population still survives^3^. Moreover, the effect of the lesion is not confined to the injured retina, since there is a microglial response in the contralateral uninjured retina^4^,^5^. Thus, this model is ideal to study *in vivo* and relatively quick the fate of axotomized neurons with or without a given treatment^3^,^4^,^5^,^6^, i.e. to test neuroprotective therapies or use genetic approaches to test whether the lack of a given protein is pernicious, protective or innocuous.

Using knock-out mice it has been reported that RGCs survive better after axotomy when the expression of pro-apoptotic genes such as Bax are ablated^7^, there is also an increase of RGCs survival upon reduction of oxidative damage^8^, inflammation^9^,^10^, nuclear atrophy^11^ or calpain activity^12^, or an activation of autophagy^13^.

In spite of these works, there is no current treatment to delay neuronal loss after traumatic injury, and therefore no effective therapies for patients. This is mainly due to the lack of knowledge about the molecular mechanism controlling neuronal death upon axotomy. However, it is known that neuroinflammation, if not the primary cause of this death, has an important role exacerbating it^14^.

Extracellular nucleotides released upon traumatic cell injury have an important role in the initiation and maintenance of the inflammatory response^15^. Extracellular nucleotides serve as an initial find-me signal for immune cell migration to the injury area and if the damage is controlled the nucleotide find-me signal promotes phagocytic clearance^16^. An increase of extracellular nucleotide concentration due to irreparable tissue damage and accumulation of other damage-associated molecular patterns leads to the activation of potent pro-inflammatory pathways by activating purinergic P2X7 receptors in innate immune cells^15^,^17^. P2X7 receptor is a cationic ion channel gated by mM concentrations of extracellular ATP, these high concentrations of ATP have been found *in vivo* associated to areas of tissue injury and inflammation^17^,^18^, and its prolonged activation leads to calcium flux, formation of large membrane pores and exposure of phosphatidylserine, ultimately resulting in cell death^19^. However, P2X7 receptor activation in the immune system can also couple to responses distinct from cell death including generation of reactive oxygen species, production of prostaglandins, release of proteases, antigen-driven T-lymphocyte proliferation, and intracellular pathogen killing^15^,^20^,^21^,^22^,^23^,^24^. P2X7 receptor is also the most potent plasma membrane receptor responsible for NLRP3 inflammasome formation and activation of caspase-1, inducing the release of pro-inflammatory cytokines of the interleukin (IL)-1 family, such as IL-1β and IL-18^21^,^25^. IL-1β is one of the main mediators of damaging during sterile inflammation and is implicated in the aetiology of many major diseases, including damage to the CNS^26^,^27^.Therefore, P2X7 receptor is a promising therapeutic target in the management of tissue damage, inflammation and pain, as witnessed by the large number of selective P2X7 receptor antagonists developed by several drug companies and currently under clinical trials^28^,^29^,^30^. In particular, A438079 is a selective competitive antagonist for the human and rodent P2X7 receptor with good bioavailability and CNS penetration widely used in preclinical animal models of disease^31^,^32^. A438079 reduces inflammation in models of colitis, ischemic acute kidney injury, contact dermatitis, and liver injury and fibrosis^33^,^34^,^35^,^36^,^37^.

P2X7 receptor is expressed in RGCs^38^,^39^,^40^ and several works suggest that it is involved in their death triggered by different insults. Thus, intravitreally injected P2X7 receptor agonists kill RGCs^41^,^42^ while its antagonism protects them in models of ocular hypertension^43^,^44^ or hypoxia^45^. Furthermore, in rats its expression increases after optic nerve axotomy^46^ and acute ocular hypertension^44^.

Therefore, we purpose to study the effect of P2X7 receptor on the survival of RGCs and the appearance of phagocytic microglial cells (PMCs) in the injured and contralateral uninjured retinas after unilateral optic nerve crush (ONC) by using the *P2rx7*^−/−^ mice and by pharmacologically targeting P2X7 receptor in wild type mice.

## Results

### P2X7 receptor expression is regulated by axotomy

**Figure 1:**
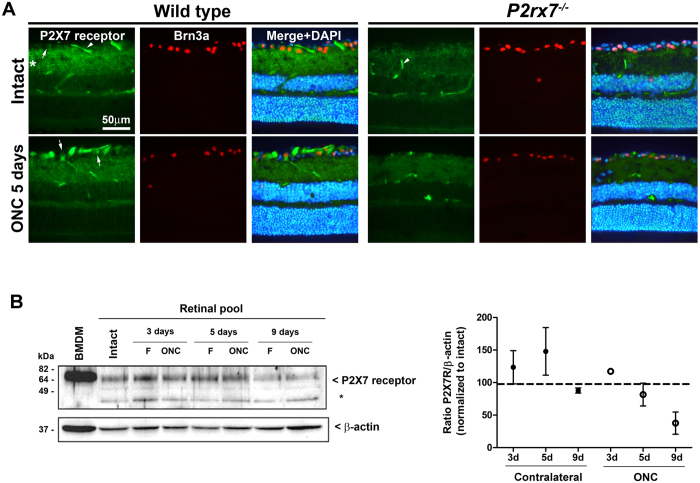
P2X7 receptor expression in the retina is regulated by optic nerve damage. (**A**) Retinal cross-sections stained for P2X7 receptor (green), Brn3a (red, detecting RGC nuclei), and nuclei (DAPI, blue), from intact or 5 days after injured (ONC) from wild type or *P2rx7*^−/−^ mice. The images show that the P2X7 receptor (green signal) in intact wild type retinas is found in the inner plexiform layer (asterisk), blood vessels (arrowheads) and weakly in the somas of RGCs (arrows, identified with Brn3a, red signal). Upon optic nerve crush, P2X7 receptor signal is stronger in the ganglion cell layer, mainly in the axons and somas of RGCs (arrows). In the *P2rx7*^−/−^ mice, some P2X7 receptor staining is observed in the inner plexiform layer and in blood vessels (arrowhead), but none in the GCL from intact or injured retinas. (**B**) Western blotting for P2X7 receptor or β-actin from cellular protein extract from mouse bone marrow derived macrophages (BMDM) or from retina pools (*n* = 3 retinas/pool) of intact retinas, injured retinas (ONC) or contralateral fellow retinas (F) at different days after ONC. Asterisk denotes unspecific protein signal found in retinas. The right panel shows densitometry quantification of two Western blots for P2X7 receptor signal normalized to β-actin and to the expression on intact retinas (dashed line). d: days. Full-length blots are presented in Supplementary Figure 1.

We first found that the P2X7 receptor was expressed in the inner plexiform layer and retinal ganglion cell layer (Fig. 1A). In intact retinas, there is a weak expression of P2X7 receptor in RGCs, in accordance with previous reports^40^,^44^. Interestingly, 5 days after ONC, there is an increase of P2X7 receptor immunostaining in the nerve fibre layer (RGC axons or astrocytes) and in the RGC somas (Fig. 1A). This signal was reduced in retinas from *P2rx7*^−/−^ animals (Fig. 1A). We also found a staining of blood vessels with the anti-P2X7 receptor antibody, but this signal was also observed in retinas from *P2rx7*^−/−^ mice, suggesting that is a non-specific signal (Fig. 1A).To confirm P2X7 receptor expression in retinas, we next performed western blotting detection in extracts from intact, injured and contralateral to the injury retinas. We found a ~75 kDa band corresponding to the size of P2X7 receptor that was also present in lysates from mouse bone marrow derived macrophages, a cell type expressing high levels of P2X7 receptor (Fig. 1B). P2X7 receptor expression in fellow retinas did not change compared to intact ones. However in the injured retinas, the expression of P2X7 receptor has a slightly increase at early time points after ONC and decreases with the time post-lesion being almost negligible at 9 days after ONC. This suggests that upon axonal injury, surviving RGCs over-express P2X7 receptor, but in the whole retina extracts its net expression decreases due to the loss of RGCs.

### Deficiency of P2X7 receptor results in a delayed loss of RGCs after axotomy

**Figure 2:**
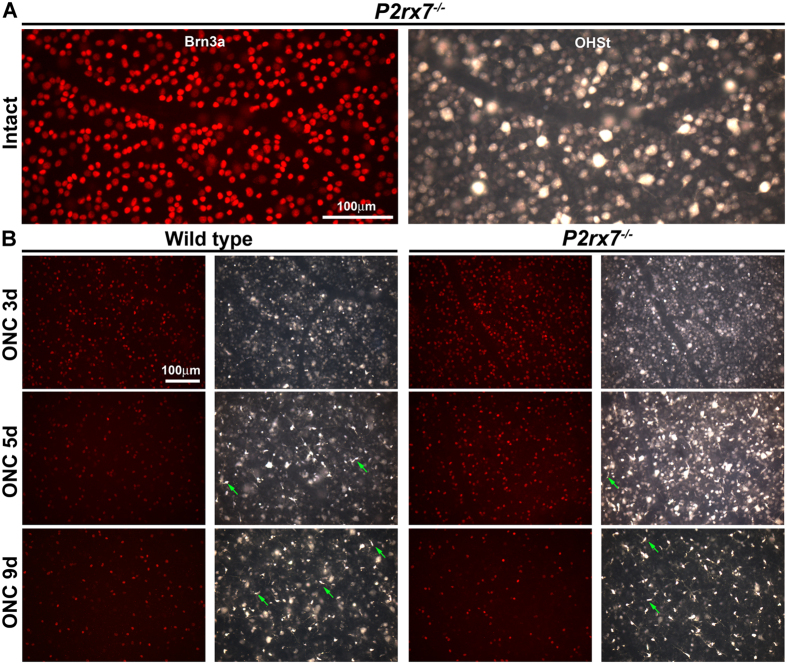
Characterization of the retinal ganglion cell population in retinas from *P2rx7*^−/−^ mice. Magnifications from flat mounted retinas showing Brn3a^+^RGCs (red) and OHSt traced-RGCs (white-blue) in the same frame from an intact *P2rx7*^−/−^ mice (**A**) and from wild type and *P2rx7*^−/−^ mice at increasing times after ONC (**B**). Green arrows point to phagocytic microglial cells. d: days.

**Figure 3:**
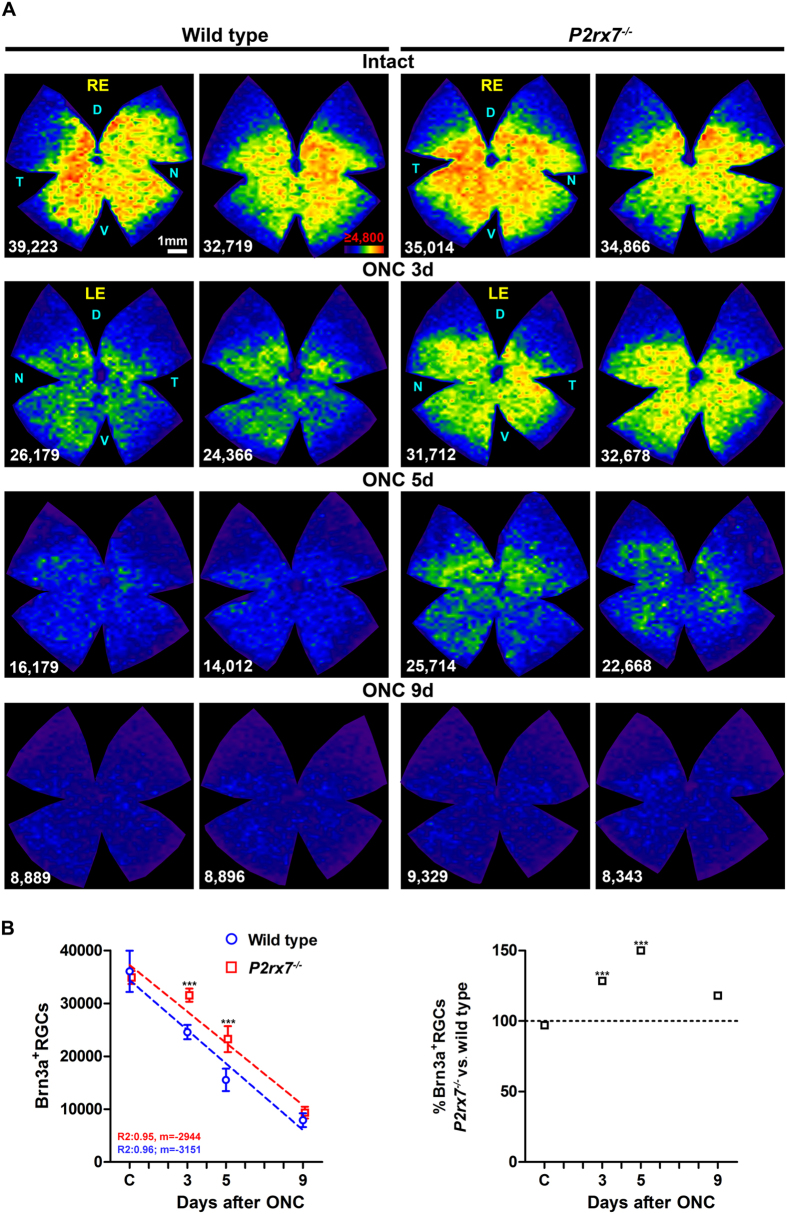
The lack of P2X7 receptor delays axotomy-induced Brn3a^+^RGC death. (**A**) Isodensity maps showing the topography of Brn3a^+^RGCs in wild type (two left columns) and *P2rx7*^−/−^ (two right columns) mice. As the time post-lesion increases, the density of Brn3a^+^RGCs decreases across the retina, but this loss is delayed in the *P2rx7*^−/−^ mouse, in accordance with the quantitative data. Colour scale for these maps is shown in the first row, second map from the left and goes from 0 (purple) RGCs/mm^2^ to 4,800 or more (red) RGCs/mm^2^. RE: right eye, LE: left injured eye. D: dorsal, N: nasal, T: temporal, V: ventral. (**B**) In the left graph the mean ± SD of Brn3a^+^RGCs in intact control (C) and injured retinas in both mice strains (Y axis) has been plotted against time post lesion (X axis). Up to 9 days after ONC, the loss of RGCs is linear and the slope of the regression line (m) is more steeper in wild type than in *P2rx7*^−/−^ mice because RGC loss is slower in the latter. The right panel shows the percentage of Brn3a^+^RGCs in the*P2rx7*^−/−^ considering 100% the number of Brn3a^+^RGCs in the wild type strain at the same time points (dashed line). *** Significantly greater number of Brn3a^+^RGCs compared to wild type at the same time point (Two way ANOVA; Bonferroni’s post-hoc test p < 0.001). d:days.

**Figure 4:**
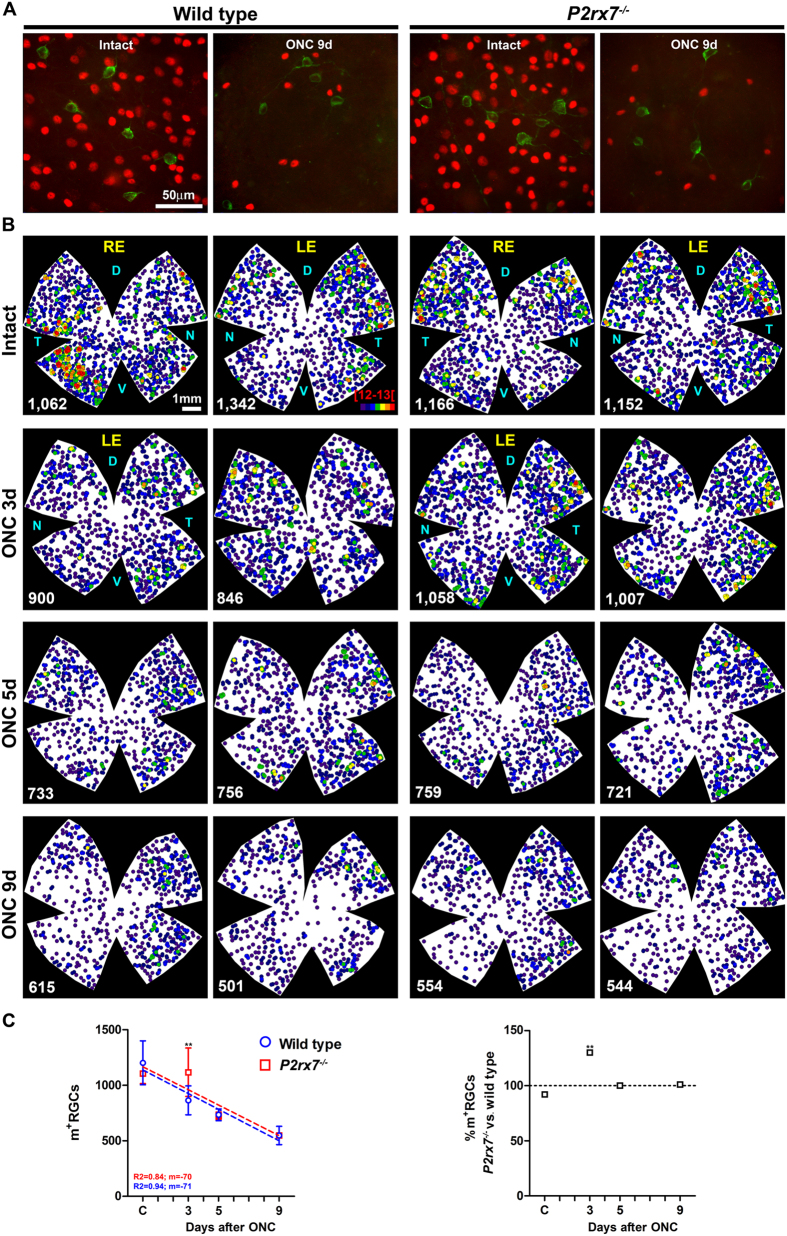
P2X7 receptor deficiency delays the loss of m^+^RGCs after axotomy. (**A**) Magnifications from flat mounted retinas showing Brn3a (red) and melanopsin (green) immunodetection in intact and injured wild type and *P2rx7*^−/−^ mice. (**B**) Neighbour maps showing the topography of m^+^RGCs in wild type (two left columns) and *P2rx7*^−/−^ (two right columns) mice. As the time post-lesion increases, the density of m^+^RGCs decreases mainly in the dorsal retina. In these maps each dot represents a single m^+^RGC and its colour indicates the number of m^+^RGCs around it (neighbours) in a radius of 0.16 mm and goes from 0–1 (purple) to 12–13 or more (red) neighbours. RE: right eye, LE: left injured eye. D: dorsal, N: nasal, T: temporal, V: ventral. (**C**) In the left graph the mean ± SD of m^+^RGCs in intact control (**C**) and injured retinas in both mice strains (Y axis) has been plotted against time post lesion (X axis). Up to 9 days after ONC, the loss of m^+^RGCs is linear but slower than the loss of RGCs. The right panel shows the percentage of m^+^RGCs in *P2rx7*^−/−^mice considering 100% the number of m^+^RGCs in the wild type strain at the same time points (dashed line). ** Significantly greater number of m^+^RGCs compared to wild type at the same time point (Two way ANOVA; Bonferroni’s post-hoc test p < 0.01). d:days.

We have analyzed in parallel the general RGC population, that transmits image-forming information to the brain and expresses the transcription factor Brn3a (Brn3a^+^RGCs)^6^,^47^,^48^,^49^ and the subpopulation of intrinsically photosensitive RGCs, that convey non-image forming information and express the photopigment melanopsin^50^,^51^ but do not express Brn3a (m^+^RGCs)^48^,^49^. The analysis of both types of RGCs is of interest because m^+^RGCs respond differently to injury and neuroprotection than the general RGC population^52^,^53^,^54^. In intact retinas, we found that *P2rx7*^−/−^ mice presented similar total number of the general RGC population identified by tracing or by Brn3a immunodetection (Fig. 2A and Table 1), as well as similar total number of m^+^RGCs when compared to wild type mice (Table 1). These data together with the topographic maps of intact retinas of both strains (Figs 3A and 4A,B), showed that the lack of the P2X7 receptor does not have an effect on the retrograde axonal transport of RGCs (tracing) nor on the number and distribution of RGCs.

In both strains the loss of Brn3a^+^RGCs occurred homogenously across the retina (Figs 2B and 3A), was exponential (RGC loss *vs*. time: R^2^ = 0.95 in wild type, R^2^ = 0.96 in *P2rx7*^−/−^. Table 2 and Fig. 3B) and significant 3 days after the lesion in the wild type strain, being delayed until day 5 in the P2X7 deficient mice (Fig. 3). Thus, in *P2rx7*^−/−^ animals the survival of RGCs is significantly higher than in wild type at 3 and 5 days after the lesion, equating at day 9. In fact, regression analysis showed that the daily loss of RGCs accounts for ~200 more RGCs in the wild type than in the deficient strain (slope of the linear regression, Fig. 3B). Topographically, we found more RGC survival throughout the retina in *P2rx7*^−/−^ mice at 3 and 5 day after injury, and the same than wild type by day 9 post-ONC (Fig. 3).

Deficiency of P2X7 receptor similarly affected to the loss of m^+^RGCs, meanwhile there was a significant lost at 3 days in wild type animals, no significant variation was observed in *P2rx7*^−/−^ retinas (Table 2 and Fig. 4B,C). However, at 5 and 9 days post-ONC the percentage of m^+^RGC survival was the same in both strains. Topographically, their loss was diffuse in both strains, but stronger in the dorsal retina (Fig. 4B).

Comparing the general RGC population (Brn3a^+^) and m^+^RGCs, we found that the percentage of survival was higher for the m^+^RGCs (Table 2), in agreement with previous reports showing in rats that m^+^RGCs are more resilient to axotomy^52^.

### P2X7 receptor antagonism in wild type animals also delays RGC loss after axotomy

**Figure 5:**
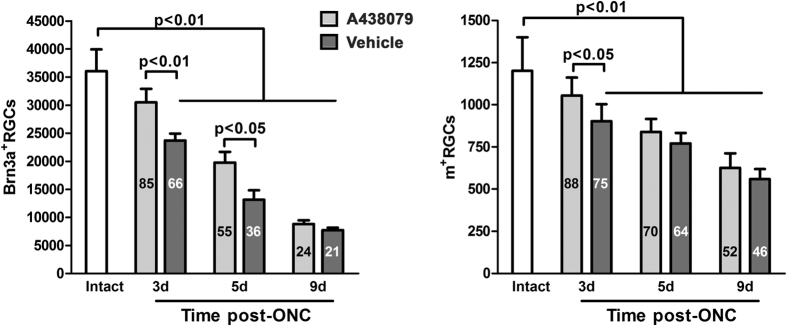
The selective P2X7 receptor antagonist A438079 increases RGC survival in wild type animals after ONC. Histograms showing the total number (mean ± standard deviation) of Brn3a^+^(left) or melanopsin^+^(right) RGCs at increasing times after ONC and a single itravitreal administration of vehicle or 300 ng of the P2X7 receptor antagonist A438079. The number inside the bars is the percentage of survival considering intact retinas as 100%. One way ANOVA with Tukey post-hoc test to compare ONC^+^vehicle or ONC^+^antagonist *vs*. intact retinas or Two way ANOVA (variables time and treatment) with Bonferroni post hoc test to compare vehicle- *vs*. antagonist- treated retinas at the same time point. d:days.

In light of the above results which indicate that the P2X7 receptor might be a potential therapeutic target for RGC loss, the next experiment was designed to study whether its antagonism was effective delaying RGC loss in the retinas of wild type mice after ONC. First, we assessed the toxicity on RGCs of an intravitreal injection of 300 ng of the selective P2X7 receptor antagonist A438079. Treated and intact retinas were analyzed 9 days after the administration, to match the longest experimental time point. The number of Brn3a^+^RGCs and of m^+^RGCs in these retinas (37,050 ± 1178 and 1197 ± 54, respectively) did not differ to that found in intact retinas (see Table 1). Hence, the antagonist appears not to be toxic for RGCs. Therefore, we administered the same dose of antagonist to ONC-injured retinas and analyzed them at 3, 5 or 9 days after the lesion. The results show that RGC loss is significantly delayed in A438079-treated retinas (Fig. 5), similarly to the delay observed in the *P2rx7*^−/−^ strain. In agreement with the quantitative data, and as observed in the P2X7 receptor deficient mice, a higher RGC survival is observed across the retina in the antagonist-treated animals (not shown).

### Phagocytic microglial cell appearance after neuronal damage

**Figure 6:**
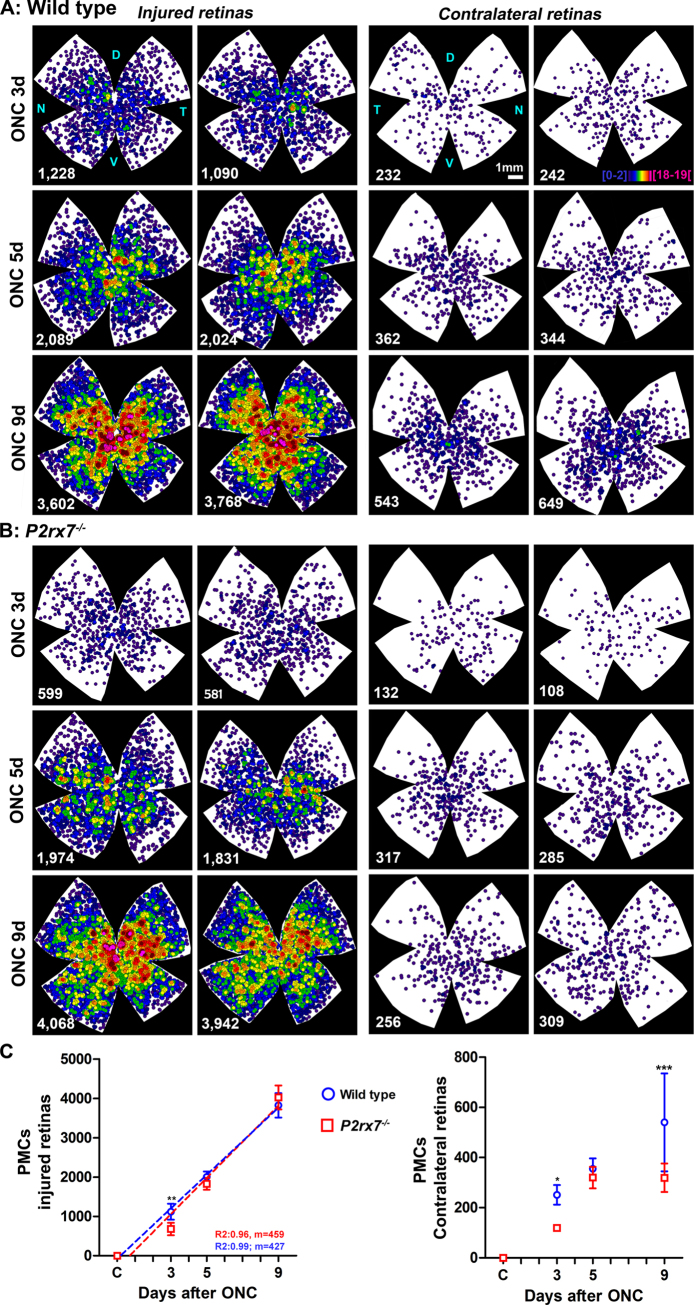
P2X7 receptor modulates phagocytic microglial cells after neuronal damage. (**A,B**) Neighbour maps depicting the distribution of phagocytic microglial cells (PMCs) in injured retinas (left retinas, two left columns) and contralateral to the injured retinas (right retinas, two right columns) in wild type (**A**) and *P2rx7*^−/−^ (**B**) mice. In these maps each dot represents a single PMC and its colour indicates the number of PMCs around it (neighbours) in a radius of 0.165 mm and goes from 0–1 (purple) to 12–13 or more (red) neighbours. D: dorsal, N: nasal, T: temporal, V: ventral. (**C**) X,Y scatter plots showing the mean ± SD of PMCs in the injured (left panel) and contralateral to the lesion (right panel) retinas of both mice strains vs. time post-lesion. In the injured retinas, the increase of PMCs is linear. *Significantly greater number of PMCs compared to wild type at the same time point (Two way ANOVA; Bonferroni’s post-hoc test * p < 0.05, **p < 0.01, ***p < 0.001). d:days.

We analyzed the appearance of phagocytic microglial cells (PMCs) measured as microglial cells that become transcellularly labelled with the tracer (OHSt) when they phagocytose a dead and traced-RGC^4^,^55^ (Table 3 and Fig. 6).

In both strains PMCs were found across the injured retina from day 3 post-lesion, and they were more abundant in the central-medial region, in the areas of higher RGC density and therefore higher RGC death (Fig. 6A,B).

As reported previously^4^, the increase of PMCs is exponential (Fig. 6C) and inversely proportional to the loss of RGCs (linear regression RGC loss *vs*. PMC appearance: R^2^ = 0.95 in wild type and R^2^ = 0.99 in the P2X7 deficient mice, regression graph not shown), i.e. their number increases as the loss of RGCs progresses (Table 3, compare left graphs in Fig. 3B -RGCs- and 5C-PMCs-). However, in *P2rx7*^−/−^ mice, there were significantly fewer PMCs 3 days after ONC when compared to injured wild type animals (Table 3), this could be a consequence to the slower RGC death found in P2X7 receptor deficient mice.

### P2X7 receptor deficiency affects the contralateral response

In a previous work, we reported that unilateral axotomy in mice caused a slight decrease of RGCs in the contralateral retina, and the appearance of PMCs^4^. Thus, we next studied RGCs and PMCs in the contralateral uninjured retinas of both strains. In wild type animals, there was a small but significant loss of RGCs after 5 days of damage that was maintained at 9 days (Table 4). In *P2rx7*^−/−^ mice, we found a delay on the loss of RGC and a significant decrease, similar to the one found in wild type animals at day 5, was observed at 9 days post-ONC (Table 4).

With respect to PMCs, in both strains and at all time points, their number in the contralateral retinas was significantly higher than in traced but otherwise intact retinas, and we found them across the retina although more concentrated in the central-medial areas (Fig. 6 and Table 4). There were, however, differences among both strains. While in wild type animals the number of PMCs increased with time, in the P2X7 deficient mice not only there were significantly fewer PMCs at 3 days than in wild type, but also their number stabilized from 5 to 9 days. (Fig. 6C and Table 4).

## Discussion

In this work we show that the lack of P2X7 receptor delays, in the injured and contralateral retina, the loss of neurons and the appearance of phagocytic microglial cells after unilateral axonal trauma. The neuroprotective effect of the specific P2X7 receptor antagonist A438079 on retinal neurons has been reported after an excitotoxic insult^56^. Here we show for the first time that also protects CNS neurons from a traumatic axonal injury.

Purinergic receptors have an important role controlling neuronal information processing and regulation of retinal tissue homeostasis (reviewed in ref. 57). In particular, expression of the ionotropic P2X7 receptor has been found in most cell types of the retina, including neurons such as the RGCs, glia cells and vascular cells^57^,^58^,^59^,^60^,^61^. However, here we found that adult *P2rx7*^−/−^ mice have a normal number of RGCs and m^+^RGCs, as well as a competent axonal transport as shown by the tracing experiments. Thus, it seems that this receptor is not necessary for neuron development and survival.

In agreement with previous reports, we show that RGCs express the P2X7 receptor, and that axotomy induces its up-regulation in the wounded neurons^44^. However, we cannot rule out that the P2X7 staining found in *P2rx7*^−/−^ retinas, especially in vascular cells, could be due to alternative splicing variants of P2X7 receptor in the *P2rx7*^−/−^ mice used in this study, as it has been already reported in this strain, P2X7 receptor variants which are predicted to escape inactivation in the brain^62^.

Prolonged P2X7 receptor stimulation induces cell death, either directly affecting the mitochondria^19^ or by inducing pyroptosis in myeloid cells after activation of the NLRP3 inflammasome and caspase-1^63^. It is reported that stimulation of P2X7 receptor can kill RGC both *in vitro* and *in vivo* by a mechanism involving a sustained increase on intracellular Ca^2+^, although the exact mechanism underlying the role of P2X7 receptor in neuronal death is to date unknown^42^,^64^. Recently, it was reported that the NLRP3 inflammasome was involved in the RGC loss after optic nerve crush injury^65^, our study suggest that P2X7 receptor, an upstream regulator of NLRP3, could be mediating the death of RGC by modulating the inflammasome.

The level of RGC protection found in the injured *P2rx7*^−/−^retinas and in the wild type retinas treated with the selective P2X7 receptor A438079 is similar to that found when administering brain derived neurotrophic factor (BDNF), to date the best described neuroprotectant^4^,^66^,^67^. Here we also show that a higher percentage of m^+^RGCs than of Brn3a^+^RGCs survive, in accordance with previous works^52^. Interestingly, while neuroprotective treatments that rescue RGCs do not seem to have an effect on m^+^RGCs^53^, we observe here that P2X7 receptor deficiency or antagonism is also beneficial for this RGC subpopulation, so P2X7 receptor signaling could be relevant in mediating m^+^RGC death.

These data are of clinical significance because several small compounds antagonizing the P2X7 receptor are being developed by the pharma industry as potential novel anti-inflammatory drugs and some of them have reached clinical trials^68^. In particular, the specific P2X7 receptor antagonism used in this study, A437089, has been satisfactory used in different preclinical models of inflammation in the periphery^33^,^34^,^35^,^36^,^37^. A438079 has been found to be CNS permeable, and its treatment reduced seizure severity during status epilepticus, as well as dopamine depletion in a model of Parkinson’s disease^69^,^70^. Furthermore, different non-specific P2X7 receptors antagonists (Brilliant Blue G and oxidized ATP) injected into the vitreous body of the eye were able to significantly preserved RGC after ONC injury^46^. Our study shows that A438079 treatment reduces RGC death, suggesting that small molecules selectively targeting P2X7 receptor could be beneficial and a novel therapeutic approach to delay neuronal cell death.

The contralateral response to unilateral injury is a well-documented effect^4^,^5^,^71^, but the underlying mechanism(s) are unknown. In adult rodents, both retinas are communicated by a very small number of RGCs that project from one retina to the other^72^,^73^,^74^. They are so few, that their loss would not account for the observed RGC loss, thus there must be additional signalling mechanisms underlying this process. Our work suggests that extracellular ATP could act as a humoral signal that reaches the contralateral retina, independent of the retino-retinal projection, however this might be difficult to explain due to the activity of ectonucleotidases in the brain that quickly degrade extracellular ATP^75^. Alternatively, P2X7 receptor signalling in the contralateral response could be related to the retino-retinal RGCs that release ATP as stress signal in the contralateral retina where their cell bodies lie upon injury of their axons that are part of the axotomized optic nerve. In line with this, the delayed loss of RGCs and PMC response in the contralateral *P2rx7*^−/−^ retina is very interesting. In wild type animals, neuroprotection of RGCs in the injured retina with BDNF does not affect the decrease of RGCs nor ameliorates the PMC response in the contralateral one^4^. Interestingly, our study shows that the deficiency of P2X7 receptor changes the contralateral response by delaying the loss of RGCs and decreasing the number of PMCs. This supports the above hypothesis stating that the contralateral response could be mediated by extracellular ATP acting as a danger signal activating the P2X7 receptor. Most probably, the slower response of microglial cells in the *P2rx7*^−/−^ retinas results from a slower rate of RGC death. However, there are also evidences showing that P2X7 receptor is required for the activation and proliferation of microglia, regulating the neuroinflammatory response and neuronal death^76^,^77^ and activation of the NLRP3 inflammasome in microglia has been found important for RGC loss after optic nerve crush injury^65^. So, even though we did not find expression of P2X7 receptor in the retinal microglia, we could not rule out the possibility that in the P2X7 receptor deficient mice, microglia function could be also impaired, for example at NLRP3 inflammasome level, and thus being the reason of a higher RGC survival.

Overall, our study suggests that after axonal injury, P2X7 receptor signal is involved in neuronal cell death, either by directly inducing death of neurons and/or by exacerbating the inflammatory response associated to tissue injury, therefore, P2X7 receptor antagonism could be a promising therapy to delay neuronal loss after traumatic injury.

## Material and Methods

### Animal handling and ethics statement

Adult pigmented C57/BL6 were obtained from the University of Murcia breeding colony and P2X7 receptor-deficient mice in a C57/BL6 background (*P2rx7*^−/−^) were purchased from Jackson^66^. Mice of 20–25 g body weight were used in this study. Animal care and experimental procedures were performed in accordance to the Association for Research in Vision and Ophthalmology, European Union guidelines for the use of animals in research and were approved by the Ethical and Animal Studies Committee of the University of Murcia (Spain).

For anaesthesia, a mixture of xylazine (10 mg/kg body weight; Rompun^®^; Bayer, Kiel, Germany) and ketamine (60 mg/kg body weight; Ketolar^®^; Pfizer, Alcobendas, Madrid, Spain) was used intraperitoneally (i.p.). After surgery, an ointment containing tobramicin (Tobrex; Alcon S.A., Barcelona, Spain) was applied on the cornea to prevent its desiccation. Mice were given oral analgesia (Buprex, Buprenorphine 0.3 mg/mL, Schering-Plough, Madrid, Spain) at 0.8 mg/kg the day of the surgery and up to the next three days.

All animals were sacrificed with an overdose of pentobarbital injected intraperitoneally (Dolethal, Vetoquinol^®^, EspecialidadesVeterinarias, S.A., Madrid, Spain).

### Surgery

*Tracing from the superior colliculi:* hydroxystilbamidinemethanesulfonate (OHSt, Molecular Probes, Leiden, The Netherland) diluted at 10% was applied to both superior colliculi one week before surgery or processing (intact animals), as previously described^4^,^6^.

*Unilateral optic nerve crush*: the left ON was crushed at 1 mm from the optic disk using previously reported methods^4^,^6^. Animals were sacrificed at increasing times post-lesion. Both retinas were analyzed, injured and contralateral to the lesion ones. Retinas from naive (intact) animals were used as control.

*Intravitreal injection* was carried out following previously described methods^4^. The left eye was injected with the P2X7 receptor selective antagonist A438079 ((3-(5-(2,3-dichlorophenyl)-1H-tetrazol-1-yl)methyl pyridine; Tocris, Bristol, United Kingdom) at a concentration of 300 ng/eye, dissolved in PBS. This dose was chosen based on previous reports^78^. The antagonist was administered to intact retinas to assess its toxicity, and to injured retinas from wild type animals right after performing ONC. The same volume of vehicle (2.5 μl) was administered in ONC-injured animals as control.

The number of retinas and the performed analyses are detailed in Table 5.

### Western blot

Retinas were fresh dissected and immediately frozen in dry ice. Then, retinas were homogenized in lysis buffer (50 mM Tris-HCl pH8.0, 150 mM NaCl, 2% Triton X-100) supplemented with 100 μl/ml of protease inhibitor mixture (Sigma-Aldrich, Madrid, Spain) for 2 min at 50 Hz using the mechanical homogenizer TissueLyser LT (Qiagen). Lysates were incubated 30 min on ice and centrifuged to remove particulate matter. Protein concentration was determined with the Bradford assay (Bio-Rad, Hercules, CA). A total of 80 μg of protein were resolved in 12% SDS-PAGE and transferred to nitrocellulose membranes (Bio-Rad, Hercules, CA) by electroblotting. Blots were blocked for 1 h with 5% skim milk in PBS containing 0.5% Tween-20 (PBS-T, pH 7.4) and then were incubated overnight at 4 °C with anti-P2X7 receptor C-terminal rabbit affinity isolated polyclonal antibody (Alomone Labs) at 1:1000 dilution followed by incubation with HRP-conjugated secondary anti-rabbit (GE Healthcare) at 1:5000 dilution. As loading control β-actin was identified using anti-β-Actin-HRP mouse monoclonal antibody (clone C4, Santa Cruz Biotechnology, Heidelberg, Germany). Membranes were developed using ECL^TM^ Prime Western Blotting Detection Reagent (GE Healthcare) and the density of the bands of proteins were quantified using image analyzer Chemidoc^TM^ XRS + (Bio-Rad, Hercules, CA) and the software (ImageLab^TM^ 5.2.1).

### Retinal dissection and immunofluorescence

Unless otherwise stated, all the reagents were from Sigma-Aldrich (Madrid, Spain). Animals were perfused transcardially with 4% paraformaldehyde (PFA) in phosphate buffer 0.1 M after a saline rinse. Then, eyes were prepared for cryosection or retinas were dissected as flat-mounts and immunofluorescence was carried out as previously reported^49^.The general population of RGCs was detected using goat anti-Brn3a (1:750, C-20, Santa Cruz Biotechnologies, Heidelberg, Germany). Melanopsin^+^RGCs (m^+^RGCs, including both M1 and M2 subtypes) were detected using rabbit anti-melanopsin UF006 antibody (1:5000 AB-N38, Advance Target Systems, ThermoFisher, Madrid, Spain) that binds to the NH_2_ terminal of the mouse melanopsin protein and thus identifies both melanopsin isoforms and detects 90% of m^+^RGCs in *Opn4*^*Cre*^*;Z/EG* reporter mice^79^. The P2X7 receptor was identified with rabbit anti-P2X7 receptor C-terminal (1:50, Alomone Labs). Secondary detection was carried out with donkey anti-goat IgG(H + L) Alexa Fluor 594 and donkey anti-rabbit IgG(H + L) Alexa Fluor 488 (1:500, Molecular Probes, ThermoFisher, Madrid, Spain). Retinal cross-sections (15 μm thick) were counterstained with DAPI (Vectashield mounting medium with DAPI, Vector laboratories, Palex Medical, Barcelona, Spain).

### Image acquisition

To make reconstructions of retinal whole-mounts (one per marker: OHSt, Brn3a and melanopsin), retinas were photographed with a x20 objective under an epifluorescence microscope (Axioscop 2 Plus; Zeiss Mikroskopie, Jena, Germany) equipped with a computer-driven motorized stage (ProScan™ H128 Series; Prior Scientific Instruments, Cambridge, UK), controlled by Image-Pro Plus (IPP 5.1 for Windows^®^; Media Cybernetics, Silver Spring, MD, USA) as previously described^6^,^49^. Individual frames were tiled to reconstruct the whole-mounts (140 individual frames/retina). Retinal cross sections were imaged with a x20 objective.

### Retinal analysis: quantification and spatial distribution

Traced- and/or Brn3a^+^RGCs were automatically quantified (image analysis software: Image-Pro Plus, IPP 5.1 for Windows; Media Cybernetics, Silver Spring, MD) using established routines by our group^3^,^4^,^6^,^49^ and their topography was visualized using isodensity maps as previously described^3^,^4^,^6^,^49^. m^+^RGCs and PMCs were manually dotted on the retinal photomontage, and the dots automatically quantified (IPP software) as described^4^,^49^. Their distribution was assessed by the fixed-radius (0.165 mm) near neighbour algorithm as reported. All maps were performed using Sigmaplot (SigmaPlot^®^ 9.0 for Windows^®^; Systat Software, Inc., Richmond, CA, EEUU).

### Statistics

Comparison of two groups (*t*-test or Mann Whitney test), more than two groups (pairwise multiple comparison procedures, ANOVA or Krustall Wallis ANOVA, and Tukey’s or Bonferroni’s post-hoc tests), the regression analysis and associated graphs were done with GraphPad Prism v 6 software (GraphPad San Diego, USA). Differences were considered significant when p < 0.05 and tests are detailed in results.

## Additional Information

**How to cite this article**: Nadal-Nicolás, F. M. *et al*. Involvement of P2X7 receptor in neuronal degeneration triggered by traumatic injury. *Sci. Rep.* **6**, 38499; doi: 10.1038/srep38499 (2016).

**Publisher’s note:** Springer Nature remains neutral with regard to jurisdictional claims in published maps and institutional affiliations.

## Supplementary Material

Supplementary Figure 1

## Figures and Tables

**Table 1:** Total number of traced-RGCs, Brn3a^+^RGCs and m^+^RGCs in intact retinas from both wild type (C57/BL6) and *P2rx7* ^−/−^ mice.

Strain		Total number of		
		OHSt^+^RGCs	Brn3a^+^RGCs	m^+^RGCs
C57/BL6 (wild type)	Mean	39,695	36,052	1,202
	SD	1,475	3,901	198
*P2rx7*^−/−^	Mean	40,983	35,159	1,106
	SD	832	1,158	91
	*p* value	*ns*	*ns*	*ns*

Values are expressed as mean ± standard deviation (SD).There was no significant difference between strains (T-test p > 0.05, *ns*).

**Table 2:** RGC survival after ONC in wild type and *P2rx7* ^−/−^ mice.

		Time after ONC (days)					
		3		5		9	
		Brn3a	Melanopsin	Brn3a	Melanopsin	Brn3a	Melanopsin
C57/BL6 (wild type)	Mean	24,556**	864*	15,522**	734**	7,912**	548**
	SD	1,346	130	2,117	53	1,311	83
	% of intact	68	71	43	61	22	46
*P2rx7*^−/−^	Mean	31,525^§§^	1,117§	23,237**^§§^	724**	9,346**	553**
	SD	1,251	218	2,440	34	1,117	87
	% of intact	90	100	66	65	26	50

Total number (mean ± standard deviation) of Brn3a^+^or m^+^RGCs at increasing times after ONC. % of intact is the percentage of surviving RGCs considering 100% the number of RGCs in intact retinas within each group.

^*^Statistically significant compared to intact retinas (One way ANOVA, Tukey post-hoc test *p < 0.01; **p < 0,001).

^§^Statistically significant compared to wild type retinas at the same time point (Two way ANOVA (variables time and strain) Bonferroni post hoc test ^§^p < 0.01^, §§^p < 0.001).

**Table 3:** Time-dynamics of phagocytic microglial cells (PMCs) in the injured retinas.

		Intact	Time after ONC (days)		
			3	5	9
C57/BL6 (wild type)	Mean	45	1,119*	2,006*	3,821*
	SD	11	203	135	310
*P2rx7*^−/−^	Mean	50	680*^§^	1,829*	4,023*
	SD	13	156	147	304

Mean number and standard deviation (SD) of phagocytic microglial cells in intact and injured retinas from wild type and *P2rx7*^−/−^ mice at different days after ONC.

^*^Significant compared to intact retinas (One way ANOVA, Tukey test p < 0.001).^§^In the knock out strain the number of PMCs was significantly lower than in wild type retinas at 3 days (Two ways ANOVA, (time and strain) Bonferroni t-test < 0.001).

**Table 4:** Contralateral response: RGCs and phagocytic microglia.

		Time after ONC (days)					
		3		5		9	
		RGCs	PMCs	RGCs	PMCs	RGCs	PMCs
C57/BL6 (wild type)	Mean	34,900	251	31,615	355	31,338	540^§^
	SD	2,422	39	2,021	41	517	195
	% of intact	97	558	88	789	87	1200
*P2rx7*^−/−^	Mean	35,476	119*	34,203	320^§^	32,162	319*
	SD	1,762	12	2,858	43	1,190	57
	% of intact	101	230	97	640	91	638

Mean number and standard deviation (SD) of RGCs and phagocytic microglial cells (PMCs) in the contralateral to the lesion retinas (right retinas) from wild type and *P2rx7*^−/−^ mice after axotomy to the left optic nerve. The percentage of RGC loss and PMC increase was calculated using as 100% the number of RGCs (Table 1) or PMCs (Table 4) in intact retinas within each strain. In wild type mice there is a significant decrease of RGCs in the right retinas 5 and 9 days after axotomizing the left optic nerve (Kruskal-Wallis One way ANOVA, Dunn’s post-hoc test p < 0.05 compared to intact retinas). A similar diminution is observed in *P2rx7*^−/−^ retinas at 9 days, but it does not reach statistical significance. At all time points and in both strains the number of PMCs in the contralateral retinas is significantly higher than in intact retinas (One way ANOVA, Tukey test p < 0.001). However, their number was significantly lower in the knock out strain at 3 and 9 days (Two ways ANOVA -time and strain-Bonferroni t-test *p < 0.001). ^§^In wild type mice, PMCs increase between 3 to 9 days while in *P2rx7*^−/−^mice the significant increase occurs between 3 and 5 days (One way ANOVA, Tukey test p ≤ 0.001).

**Table 5:** Number of retinas analyzed in this study.

Wild type vs. *P2rx7*^−/−^			C57/BL6 (wild type)			*P2rx7*^−/−^		
			Quantification		WB	Quantification		WB
			RGCs	PMCs		RGCs	PMCs	
Intact			4	4	4	6	4	4
Unilateral ONC	3d	Injured	7	4	4	4	3	4
		Contralateral	7	6	4	4	3	4
	5d	Injured	7	4	4	6	3	4
		Contralateral	7	6	4	6	6	4
	9d	Injured	6	4	4	7	4	4
		Contralateral	6	6	4	7	6	4
**P2X7 receptor antagonism**	9d		**Quantification RGCs**					
Intact + A438079 (toxicity)			4					
ONC + A438079 or vehicle	3d	A438079	4					
		Vehicle	4					
	5d	A438079	4					
		Vehicle	4					
	9d	A438079	4					
		Vehicle	4					

In addition, 2 intact eyes, and 2 eyes/strain processed 5 days after ONC were used for cross-sections. WB: western blotting. PMC: phagocytic microglial cells, RGCs: retinal ganglion cells.

## References

[b1] Vidal-Sanz M. . Retinal neurodegeneration in experimental glaucoma. Prog. Brain Res. 220, 1–35 (2015). 26497783 10.1016/bs.pbr.2015.04.008

[b2] Tzekov R. . Sub-Chronic Neuropathological and Biochemical Changes in Mouse Visual System after Repetitive Mild Traumatic Brain Injury. PLoS. One. 11(4), e0153608 (2016). 27088355 10.1371/journal.pone.0153608 PMC4835061

[b3] Sanchez-Migallon M. C., Valiente-Soriano F. J., Nadal-Nicolas F. M., Vidal-Sanz M. & Agudo-Barriuso M. Apoptotic Retinal Ganglion Cell Death After Optic Nerve Transection or Crush in Mice: Delayed RGC Loss With BDNF or a Caspase 3 Inhibitor. Invest Ophthalmol. Vis. Sci 57(1), 81–93 (2016). 26780312 10.1167/iovs.15-17841

[b4] Galindo-Romero C. . Effect of brain-derived neurotrophic factor on mouse axotomized retinal ganglion cells and phagocytic microglia. Invest Ophthalmol. Vis. Sci 54(2), 974–985 (2013). 23307961 10.1167/iovs.12-11207

[b5] Ramirez A. I. . Macro- and microglial responses in the fellow eyes contralateral to glaucomatous eyes. Prog. Brain Res. 220, 155–172 (2015). 26497789 10.1016/bs.pbr.2015.05.003

[b6] Galindo-Romero C. . Axotomy-induced retinal ganglion cell death in adult mice: quantitative and topographic time course analyses. Exp. Eye Res. 92(5), 377–387 (2011). 21354138 10.1016/j.exer.2011.02.008

[b7] Goldenberg-Cohen N., Dratviman-Storobinsky O., Dadon Bar E. S., Cheporko Y. & Hochhauser E. Protective effect of Bax ablation against cell loss in the retinal ganglion layer induced by optic nerve crush in transgenic mice. J. Neuroophthalmol. 31(4), 331–338 (2011). 21799446 10.1097/WNO.0b013e318227e4fb

[b8] Xiong W., MacColl Garfinkel A. E., Li Y., Benowitz L. I. & Cepko C. L. NRF2 promotes neuronal survival in neurodegeneration and acute nerve damage. J. Clin. Invest 125(4), 1433–1445 (2015). 25798616 10.1172/JCI79735 PMC4396467

[b9] Mac Nair C. E., Fernandes K. A., Schlamp C. L., Libby R. T. & Nickells R. W. Tumor necrosis factor alpha has an early protective effect on retinal ganglion cells after optic nerve crush. J. Neuroinflammation. 11, 194 (2014). 25407441 10.1186/s12974-014-0194-3 PMC4245774

[b10] Tezel G., Yang X., Yang J. & Wax M. B. Role of tumor necrosis factor receptor-1 in the death of retinal ganglion cells following optic nerve crush injury in mice. Brain Res. 996(2), 202–212 (2004). 14697498 10.1016/j.brainres.2003.10.029

[b11] Schmitt H. M., Pelzel H. R., Schlamp C. L. & Nickells R. W. Histone deacetylase 3 (HDAC3) plays an important role in retinal ganglion cell death after acute optic nerve injury. Mol. Neurodegener. 9, 39 (2014). 25261965 10.1186/1750-1326-9-39 PMC4190472

[b12] Ryu M. . Critical role of calpain in axonal damage-induced retinal ganglion cell death. J. Neurosci. Res. 90(4), 802–815 (2012). 22065590 10.1002/jnr.22800

[b13] Rodriguez-Muela N., Germain F., Marino G., Fitze P. S. & Boya P. Autophagy promotes survival of retinal ganglion cells after optic nerve axotomy in mice. Cell Death. Differ. 19(1), 162–169 (2012). 21701497 10.1038/cdd.2011.88 PMC3252838

[b14] Schuetz E. & Thanos S. Microglia-targeted pharmacotherapy in retinal neurodegenerative diseases. Curr. Drug Targets. 5(7), 619–627 (2004). 15473251 10.2174/1389450043345164

[b15] Idzko M., Ferrari D. & Eltzschig H. K. Nucleotide signalling during inflammation. Nature 509(7500), 310–317 (2014). 24828189 10.1038/nature13085 PMC4222675

[b16] Elliott M. R. . Nucleotides released by apoptotic cells act as a find-me signal to promote phagocytic clearance. Nature 461(7261), 282–286 (2009). 19741708 10.1038/nature08296 PMC2851546

[b17] Barbera-Cremades M. . P2X7 receptor-stimulation causes fever via PGE2 and IL-1beta release. FASEB J. 26(7), 2951–2962 (2012). 22490780 10.1096/fj.12-205765

[b18] Wilhelm K. . Graft-versus-host disease is enhanced by extracellular ATP activating P2X7R. Nat. Med. 16(12), 1434–1438 (2010). 21102458 10.1038/nm.2242

[b19] Mackenzie A. B., Young M. T., Adinolfi E. & Surprenant A. Pseudoapoptosis induced by brief activation of ATP-gated P2X7 receptors. J. Biol. Chem. 280(40), 33968–33976 (2005). 15994333 10.1074/jbc.M502705200

[b20] Coutinho-Silva R. . Inhibition of chlamydial infectious activity due to P2X7R-dependent phospholipase D activation. Immunity. 19(3), 403–412 (2003). 14499115 10.1016/s1074-7613(03)00235-8

[b21] Ferrari D. . The P2X7 receptor: a key player in IL-1 processing and release. J. Immunol. 176(7), 3877–3883 (2006). 16547218 10.4049/jimmunol.176.7.3877

[b22] Lopez-Castejon G. . P2X(7) receptor-mediated release of cathepsins from macrophages is a cytokine-independent mechanism potentially involved in joint diseases. J. Immunol. 185(4), 2611–2619 (2010). 20639492 10.4049/jimmunol.1000436

[b23] Moore S. F. & Mackenzie A. B. NADPH oxidase NOX2 mediates rapid cellular oxidation following ATP stimulation of endotoxin-primed macrophages. J. Immunol. 183(5), 3302–3308 (2009). 19696433 10.4049/jimmunol.0900394

[b24] Yip L. . Autocrine regulation of T-cell activation by ATP release and P2X7 receptors. FASEB J. 23(6), 1685–1693 (2009). 19211924 10.1096/fj.08-126458 PMC2718802

[b25] Pelegrin P., “Inflammasome Activation by Danger Signals,“in The Inflammasomes edited by Couillin I., Pertrilli V. & Martinon F. pp.101–121 (Springer, Basel, Switzerland, 2011).

[b26] Denes A. . AIM2 and NLRC4 inflammasomes contribute with ASC to acute brain injury independently of NLRP3. Proc. Natl. Acad. Sci USA 112(13), 4050–4055 (2015). 25775556 10.1073/pnas.1419090112 PMC4386342

[b27] Galea J. & Brough D. The role of inflammation and interleukin-1 in acute cerebrovascular disease. J. Inflamm. Res. 6, 121–128 (2013). 24062616 10.2147/JIR.S35629 PMC3780292

[b28] Arulkumaran N., Unwin R. J. & Tam F. W. A potential therapeutic role for P2X7 receptor (P2X7R) antagonists in the treatment of inflammatory diseases. Expert. Opin. Investig. Drugs 20(7), 897–915 (2011). 10.1517/13543784.2011.578068 PMC3114873 21510825

[b29] King B. F. Novel P2X7 receptor antagonists ease the pain. Br. J. Pharmacol. 151(5), 565–567 (2007). 17471176 10.1038/sj.bjp.0707266 PMC2013988

[b30] Pelegrin P. Targeting interleukin-1 signaling in chronic inflammation: focus on P2X(7) receptor and Pannexin-1. Drug News Perspect. 21(8), 424–433 (2008). 19034348 10.1358/dnp.2008.21.8.1265800

[b31] Nelson D. W. . Structure-activity relationship studies on a series of novel, substituted 1-benzyl-5-phenyltetrazole P2X7 antagonists. J. Med. Chem. 49(12), 3659–3666 (2006). 16759108 10.1021/jm051202e

[b32] McGaraughty S. . P2X7-related modulation of pathological nociception in rats. Neuroscience 146(4), 1817–1828 (2007). 17478048 10.1016/j.neuroscience.2007.03.035

[b33] Wan P. . Extracellular ATP mediates inflammatory responses in colitis via P2 x 7 receptor signaling. Sci Rep. 6, 19108 (2016). 26739809 10.1038/srep19108 PMC4703960

[b34] Yan Y. . P2X7 receptor inhibition protects against ischemic acute kidney injury in mice. Am. J. Physiol Cell Physiol 308(6), C463–C472 (2015). 25588875 10.1152/ajpcell.00245.2014 PMC4360025

[b35] Zanin R. F. . Decrease of serum adenine nucleotide hydrolysis in an irritant contact dermatitis mice model: potential P2X7R involvement. Mol. Cell Biochem. 404 **(1–2)**, 221–228 (2015). 25772484 10.1007/s11010-015-2381-7

[b36] Huang C. . P2X7 blockade attenuates mouse liver fibrosis. Mol. Med. Rep. 9(1), 57–62 (2014). 24247209 10.3892/mmr.2013.1807

[b37] Xie Y. . Purinergic receptor antagonist A438079 protects against acetaminophen-induced liver injury by inhibiting p450 isoenzymes, not by inflammasome activation. Toxicol. Sci 131(1), 325–335 (2013). 22986947 10.1093/toxsci/kfs283 PMC3537131

[b38] Franke H. . P2X(7) receptor-mRNA and -protein in the mouse retina; changes during retinal degeneration in BALBCrds mice. Neurochem. Int. 47(4), 235–242 (2005). 15964665 10.1016/j.neuint.2005.04.022

[b39] Niyadurupola N. . P2X7 receptor activation mediates retinal ganglion cell death in a human retina model of ischemic neurodegeneration. Invest Ophthalmol. Vis. Sci 54(3), 2163–2170 (2013). 23449724 10.1167/iovs.12-10968

[b40] Wheeler-Schilling T. H., Marquordt K., Kohler K., Guenther E. & Jabs R. Identification of purinergic receptors in retinal ganglion cells. Brain Res. Mol. Brain Res. 92(1–2), 177–180 (2001). 11483255 10.1016/s0169-328x(01)00160-7

[b41] Resta V. . Acute retinal ganglion cell injury caused by intraocular pressure spikes is mediated by endogenous extracellular ATP. Eur. J. NeuroSci. 25(9), 2741–2754 (2007). 17459106 10.1111/j.1460-9568.2007.05528.x

[b42] Zhang X., Zhang M., Laties A. M. & Mitchell C. H. Stimulation of P2X7 receptors elevates Ca2 + and kills retinal ganglion cells. Invest Ophthalmol. Vis. Sci 46(6), 2183–2191 (2005). 15914640 10.1167/iovs.05-0052

[b43] Mitchell C. H. . The P2X(7) receptor in retinal ganglion cells: A neuronal model of pressure-induced damage and protection by a shifting purinergic balance. Purinergic. Signal. 5(2), 241–249 (2009). 19241145 10.1007/s11302-009-9142-6 PMC2686831

[b44] Sugiyama T. . P2X(7) receptor activation may be involved in neuronal loss in the retinal ganglion cell layer after acute elevation of intraocular pressure in rats. Mol. Vis. 19, 2080–2091 (2013). 24146541 PMC3786454

[b45] Sugiyama T. . Involvement of P2X7 receptors in the hypoxia-induced death of rat retinal neurons. Invest Ophthalmol. Vis. Sci 51(6), 3236–3243 (2010). 20071682 10.1167/iovs.09-4192

[b46] Kakurai K., Sugiyama T., Kurimoto T., Oku H. & Ikeda T. Involvement of P2X(7) receptors in retinal ganglion cell death after optic nerve crush injury in rats. Neurosci. Lett. 534, 237–241 (2013). 23262079 10.1016/j.neulet.2012.11.060

[b47] Nadal-Nicolas F. M. . Whole number, distribution and co-expression of brn3 transcription factors in retinal ganglion cells of adult albino and pigmented rats. PLoS. One. 7(11), e49830 (2012). 23166779 10.1371/journal.pone.0049830 PMC3500320

[b48] Nadal-Nicolas F. M. . Displaced retinal ganglion cells in albino and pigmented rats. Front Neuroanat. 8, 99 (2014). 25339868 10.3389/fnana.2014.00099 PMC4186482

[b49] Valiente-Soriano F. J. . Distribution of melanopsin positive neurons in pigmented and albino mice: evidence for melanopsin interneurons in the mouse retina. Front Neuroanat. 8, 131 (2014). 25477787 10.3389/fnana.2014.00131 PMC4238377

[b50] Provencio I. . A novel human opsin in the inner retina. J. Neurosci. 20(2), 600–605 (2000). 10632589 10.1523/JNEUROSCI.20-02-00600.2000 PMC6772411

[b51] Schmidt T. M., Chen S. K. & Hattar S. Intrinsically photosensitive retinal ganglion cells: many subtypes, diverse functions. Trends Neurosci. 34(11), 572–580 (2011). 21816493 10.1016/j.tins.2011.07.001 PMC3200463

[b52] Nadal-Nicolas F. M., Sobrado-Calvo P., Jimenez-Lopez M., Vidal-Sanz M. & Agudo-Barriuso M. Long-Term Effect of Optic Nerve Axotomy on the Retinal Ganglion Cell Layer. Invest Ophthalmol. Vis. Sci 56(10), 6095–6112 (2015). 26393669 10.1167/iovs.15-17195

[b53] Valiente-Soriano F. J. . BDNF Rescues RGCs But Not Intrinsically Photosensitive RGCs in Ocular Hypertensive Albino Rat Retinas. Invest Ophthalmol. Vis. Sci 56(3), 1924–1936 (2015). 25722208 10.1167/iovs.15-16454

[b54] Valiente-Soriano F. J. . Effects of ocular hypertension in the visual system of pigmented mice. PLoS. One. 10(3), e0121134 (2015). 25811653 10.1371/journal.pone.0121134 PMC4374934

[b55] Thanos S. Specific transcellular carbocyanine-labelling of rat retinal microglia during injury-induced neuronal degeneration. Neurosci. Lett. 127(1), 108–112 (1991). 1881605 10.1016/0304-3940(91)90906-a

[b56] Sakamoto K. . P2X7 receptor antagonists protect against N-methyl-D-aspartic acid-induced neuronal injury in the rat retina. Eur. J. Pharmacol. 756, 52–58 (2015). 25796199 10.1016/j.ejphar.2015.03.008

[b57] Reichenbach A. & Bringmann A. Purinergic signaling in retinal degeneration and regeneration. Neuropharmacology 104, 194–211 (2015). 25998275 10.1016/j.neuropharm.2015.05.005

[b58] Brandle U., Kohler K. & Wheeler-Schilling T. H. Expression of the P2X7-receptor subunit in neurons of the rat retina. Brain Res. Mol. Brain Res. 62(1), 106–109 (1998). 9795168 10.1016/s0169-328x(98)00254-x

[b59] Ishii K., Kaneda M., Li H., Rockland K. S. & Hashikawa T. Neuron-specific distribution of P2X7 purinergic receptors in the monkey retina. J. Comp Neurol. 459(3), 267–277 (2003). 12655509 10.1002/cne.10608

[b60] Kawamura H. . ATP: a vasoactive signal in the pericyte-containing microvasculature of the rat retina. J. Physiol 551 (Pt 3), 787–799 (2003). 12876212 10.1113/jphysiol.2003.047977 PMC2343299

[b61] Morigiwa K., Quan M., Murakami M., Yamashita M. & Fukuda Y. P2 Purinoceptor expression and functional changes of hypoxia-activated cultured rat retinal microglia. Neurosci. Lett. 282(3), 153–156 (2000). 10717414 10.1016/s0304-3940(00)00887-9

[b62] Masin M. . Expression, assembly and function of novel C-terminal truncated variants of the mouse P2X7 receptor: re-evaluation of P2X7 knockouts. Br. J. Pharmacol. 165(4), 978–993 (2012). 21838754 10.1111/j.1476-5381.2011.01624.x PMC3312493

[b63] Martin-Sanchez F. . Inflammasome-dependent IL-1beta release depends upon membrane permeabilisation. Cell Death. Differ. 23(7), 1219–31 (2016). 26868913 10.1038/cdd.2015.176 PMC4946890

[b64] Innocenti B., Pfeiffer S., Zrenner E., Kohler K. & Guenther E. ATP-induced non-neuronal cell permeabilization in the rat inner retina. J. Neurosci. 24(39), 8577–8583 (2004). 15456831 10.1523/JNEUROSCI.2812-04.2004 PMC6729894

[b65] Puyang Z. . Retinal Ganglion Cell Loss is Delayed Following Optic Nerve Crush in NLRP3 Knockout Mice. Sci Rep. 6, 20998 (2016). 26893104 10.1038/srep20998 PMC4759563

[b66] Di Polo A., Aigner L. J., Dunn R. J., Bray G. M. & Aguayo A. J. Prolonged delivery of brain-derived neurotrophic factor by adenovirus-infected Muller cells temporarily rescues injured retinal ganglion cells. Proc. Natl. Acad. Sci USA 95(7), 3978–3983 (1998). 9520478 10.1073/pnas.95.7.3978 PMC19948

[b67] Sanchez-Migallon M. C. . Brain derived neurotrophic factor maintains Brn3a expression in axotomized rat retinal ganglion cells. Exp. Eye Res. 92(4), 260–267 (2011). 21315070 10.1016/j.exer.2011.02.001

[b68] Lopez-Castejon G. & Pelegrin P. Current status of inflammasome blockers as anti-inflammatory drugs. Expert. Opin. Investig. Drugs 21(7), 995–1007 (2012). 10.1517/13543784.2012.690032 22612568

[b69] Jimenez-Pacheco A. . Increased neocortical expression of the P2X7 receptor after status epilepticus and anticonvulsant effect of P2X7 receptor antagonist A-438079. Epilepsia 54(9), 1551–1561 (2013). 23808395 10.1111/epi.12257

[b70] Marcellino D. . On the role of P2X(7) receptors in dopamine nerve cell degeneration in a rat model of Parkinson’s disease: studies with the P2X(7) receptor antagonist A-438079. J. Neural Transm. (Vienna.) 117(6), 681–687 (2010). 20387084 10.1007/s00702-010-0400-0

[b71] Bodeutsch N., Siebert H., Dermon C. & Thanos S. Unilateral injury to the adult rat optic nerve causes multiple cellular responses in the contralateral site. J. Neurobiol. 38(1), 116–128 (1999). 10027567 10.1002/(sici)1097-4695(199901)38:1<116::aid-neu9>3.0.co;2-f

[b72] Nadal-Nicolas F. M. . Retino-retinal projection in juvenile and young adult rats and mice. Exp. Eye Res. 134, 47–52 (2015). 25797477 10.1016/j.exer.2015.03.015

[b73] Avellaneda-Chevrier V. K., Wang X., Hooper M. L. & Chauhan B. C. The retino-retinal projection: Tracing retinal ganglion cells projecting to the contralateral retina. Neurosci. Lett. 591, 105–109 (2015). 25700948 10.1016/j.neulet.2015.02.033

[b74] Tang X., Tzekov R. & Passaglia C. L. Retinal crosstalk in the mammalian visual system. J. Neurophysiol. 115(6), 3018–29 (2016). 26984426 10.1152/jn.01137.2015 PMC4946590

[b75] Robson S. C., Sevigny J. & Zimmermann H. The E-NTPDase family of ectonucleotidases: Structure function relationships and pathophysiological significance. Purinergic. Signal. 2(2), 409–430 (2006). 18404480 10.1007/s11302-006-9003-5 PMC2254478

[b76] Monif M., Reid C. A., Powell K. L., Smart M. L. & Williams D. A. The P2X7 receptor drives microglial activation and proliferation: a trophic role for P2X7R pore. J. Neurosci. 29(12), 3781–3791 (2009). 19321774 10.1523/JNEUROSCI.5512-08.2009 PMC6665035

[b77] Skaper S. D. . P2X(7) receptors on microglial cells mediate injury to cortical neurons *in vitro*. Glia 54(3), 234–242 (2006). 16817206 10.1002/glia.20379

[b78] Mizutani T. . Nucleoside Reverse Transcriptase Inhibitors Suppress Laser-Induced Choroidal Neovascularization in Mice. Invest Ophthalmol. Vis. Sci 56(12), 7122–7129 (2015). 26529046 10.1167/iovs.15-17440 PMC4634627

[b79] Brown T. M. . Melanopsin contributions to irradiance coding in the thalamo-cortical visual system. PLoS. Biol. 8(12), e1000558 (2010). 21151887 10.1371/journal.pbio.1000558 PMC2998442

