# Supplementary material for: Involvement of P2X7 receptor in neuronal degeneration triggered by traumatic injury
Source: Sci Rep. 2016 Dec 8;6:38499. doi: 10.1038/srep38499 (PMC5144087; doi:10.1038/srep38499)
Supplement: Supplementary Figure 1 [file srep38499-s1.pdf]

Involvement of P2X7 receptor in neuronal degeneration triggered by traumatic injury

Nadal-Nicolás, FM., Galindo-Romero, MC., Valiente-Soriano, FJ., Barberá-Cremades, M., deTorre-Minguela, C., Salinas-Navarro, M. , Pelegrín, P., Agudo-Barriuso, M

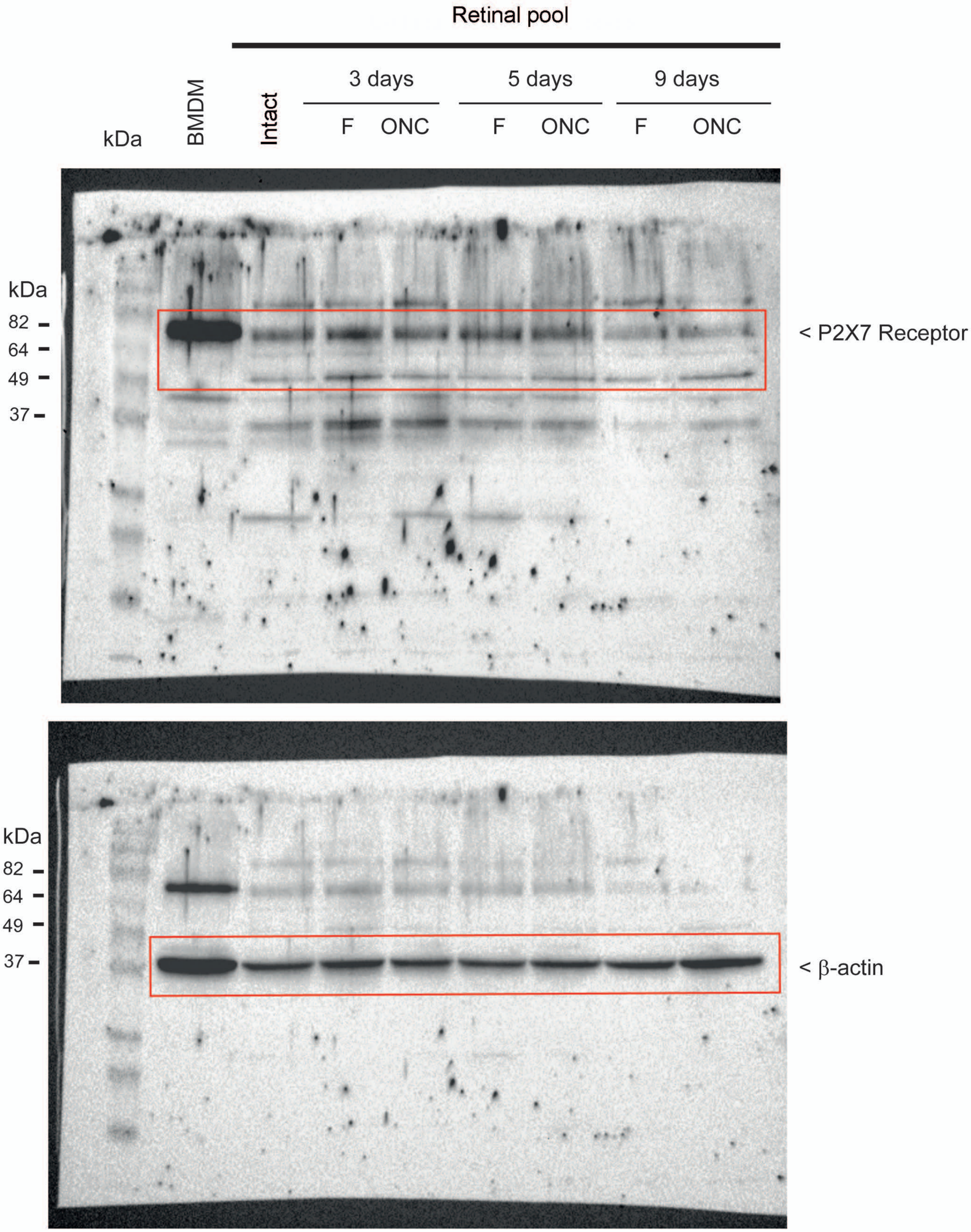

Supplementary figure 1
